# Stereoselectivity in Cell Uptake by SLC22 Organic Cation Transporters 1, 2, and 3

**DOI:** 10.1021/acs.jmedchem.3c01436

**Published:** 2023-12-05

**Authors:** Lukas Gebauer, Ole Jensen, Muhammad Rafehi, Jürgen Brockmöller

**Affiliations:** Institute of Clinical Pharmacology, University Medical Center Göttingen, Göttingen D-37075, Germany

## Abstract

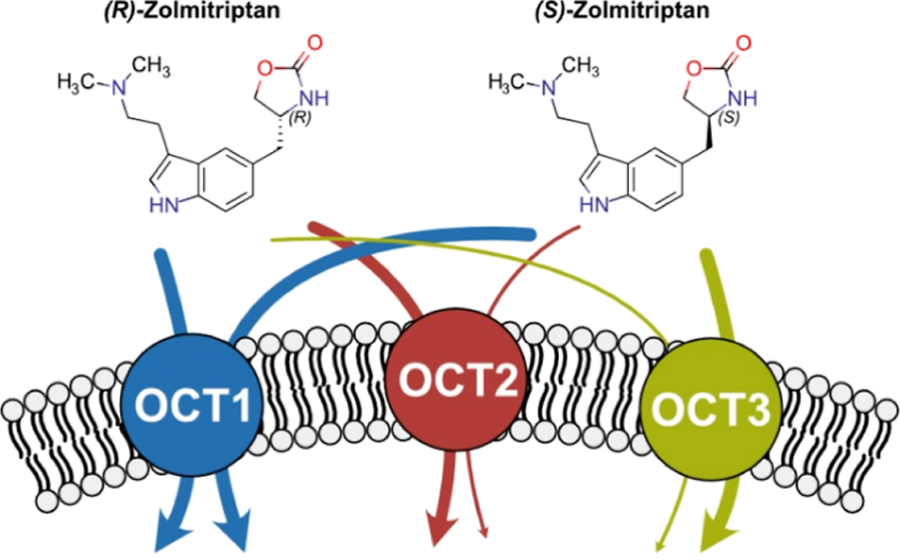

Stereoselectivity can be most relevant in drug metabolism and receptor binding. Although drug membrane transport might be equally important for small-molecule pharmacokinetics, the extent of stereoselectivity in membrane transport is largely unknown. Here, we characterized the stereoselective transport of 18 substrates of SLC22 organic cation transporters (OCTs) 1, 2, and 3. OCT2 and OCT3 showed highly stereoselective cell uptake with several substrates and, interestingly, often with opposite stereoselectivity. In contrast, transport by OCT1 was less stereoselective, although (*R*)-tamsulosin was transported by OCT1 with higher apparent affinity than the (*S*)-enantiomer. Using OCT1 and CYP2D6 co-overexpressing cells, an additive effect of the stereoselectivities was demonstrated. This indicates that pharmacokinetic stereoselectivity may be the result of combined effects in transport and metabolism. This study highlights that the pronounced polyspecificity of OCTs not contradicts stereoselectivity in the transport. Nevertheless, stereoselectivity is highly substrate-specific and for most substrates and OCTs, there was no major selectivity.

## Introduction

The majority of all drugs are chiral, and at present, most of them are therapeutically applied as racemates, the equimolar mixture of two complementary enantiomers. Although most chemical properties of enantiomers are identical, they frequently exhibit different biological activities due to stereoselective interactions with proteins composed of homochiral amino acids. In pharmacotherapy, it is well established that drug receptor binding^1,2^ and drug metabolism^3^ often may be highly stereoselective. Even though drug membrane transport might be of similar importance for the pharmacokinetics of small molecules, less is known about stereoselectivity of membrane transporters.

The organic cation transporters 1, 2, and 3 (OCT1–3, *SLC22A1–3*) are membrane transporter proteins with a broad substrate selectivity.^4^ OCT1 and OCT2 share about 70% amino acid identity,^5^ whereas OCT3 has a sequence homology of 50% toward both.^6^ OCT1 and OCT2 have dominant hepatic or renal expression,^7,8^ respectively, whereas OCT3 has a more broad profile of expression and is expressed, for instance, in the heart,^9^ the brain,^10^ and at the blood–brain barrier.^11^ A prominent feature, especially for OCT1, is its high genetic variability. The inherited *SLC22A1* genetic polymorphisms are found with a reasonable frequency in many populations.^12^ The functional consequences range from a reduced function to a complete loss of function and may be relevant for the pharmacokinetics of several drugs.^13−15^ OCT2 has one frequent polymorphism (Ala270Ser) which only leads to moderate impairment of transporter function.^16^ Also in OCT3, several heritable variants have been recently described and are possibly associated with psychiatric diseases.^17^ Although this may highlight the role of OCT3 in brain cation homeostasis, the allele frequencies of these variants are probably too low to have broad, population-relevant pharmacokinetic effects.

**Figure 1:**
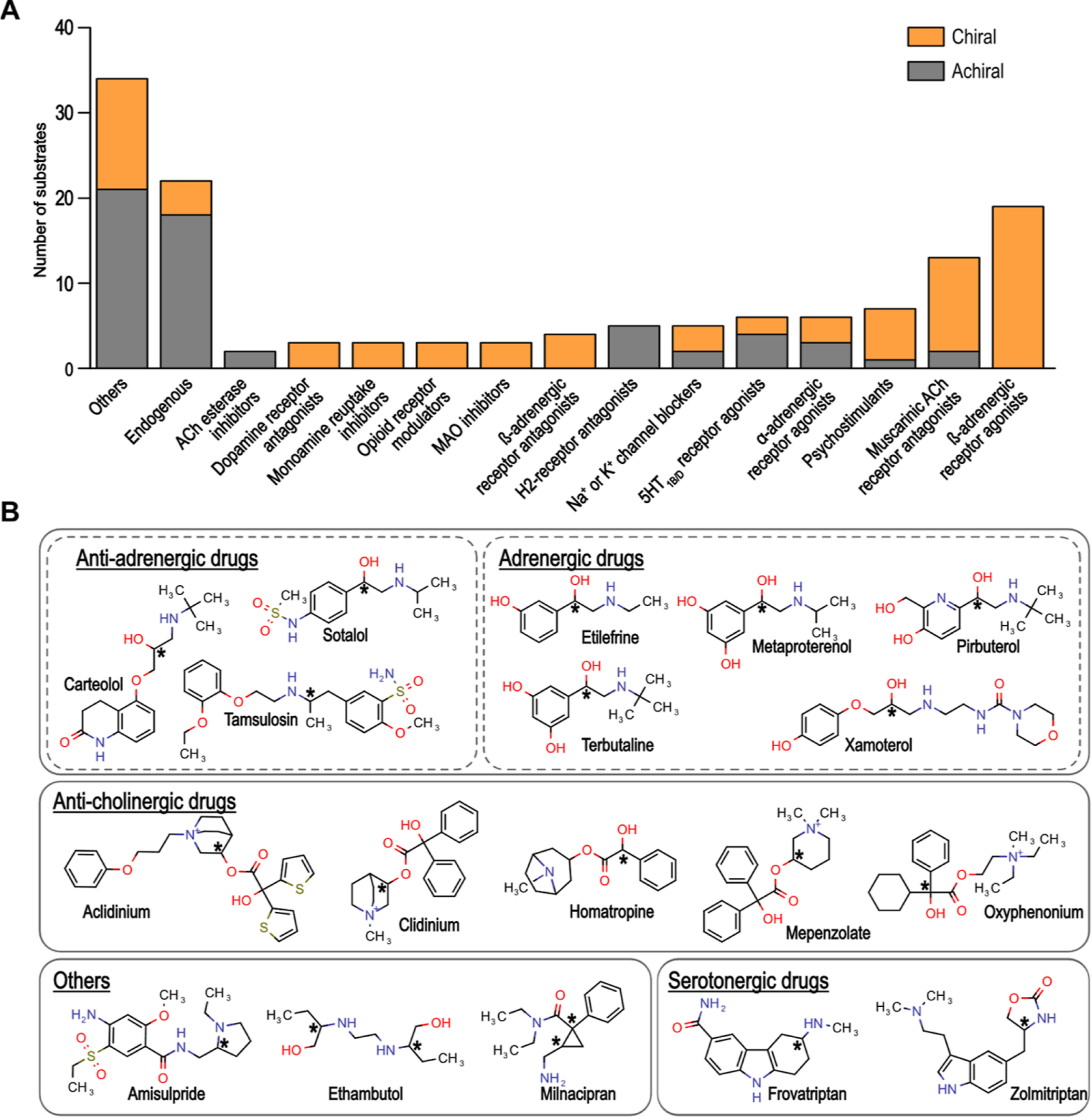
Chirality in therapeutic drug classes depending on cell uptake by SLC22 organic cation transporters. Overview of known OCT substrates classified according to their drug class (A). The orange filling of each box indicates the respective proportions of chiral drugs. These OCT substrates were identified earlier.^19−25^ Overview of substrates investigated in this study (B). Chiral centers are highlighted with asterisks.

OCTs transport hydrophilic, mainly (but not exclusively^18^) positively charged substances with low to moderate molecular weight between 150 and 450 Da.^19−21^ Drugs of different therapeutic areas are transported by OCTs. Most prominent are drugs acting as β-adrenergic receptor agonists or muscarinic acetylcholine receptor antagonists (Figure 1A).

Both classes are composed almost completely of chiral molecules. In earlier studies, stereoselectivity in the transport by OCTs has only been investigated for (anti)adrenergic drugs as well as for chiral phenylethylamines which are structurally related to adrenergic drugs.^26−28^ For a more comprehensive characterization, we extended this series by investigating the cellular uptake of additional (anti)adrenergic substrates but also included several anticholinergic substances among other OCT substrates (Figure 1B). All substances are known OCT substrates, but stereoselectivity in their transport was unknown. Moreover, we analyzed whether the most common, functionally relevant polymorphisms of OCT1 (Met420del and Arg61Cys) and OCT2 (Ala270Ser) affect stereoselectivity. Finally, we extended the scope of this study by investigating in a few examples the combined stereoselectivities of OCT1 and the drug-metabolizing enzyme cytochrome P450 2D6 on the cellular disposition of shared substrates.

## Results

**Figure 2:**
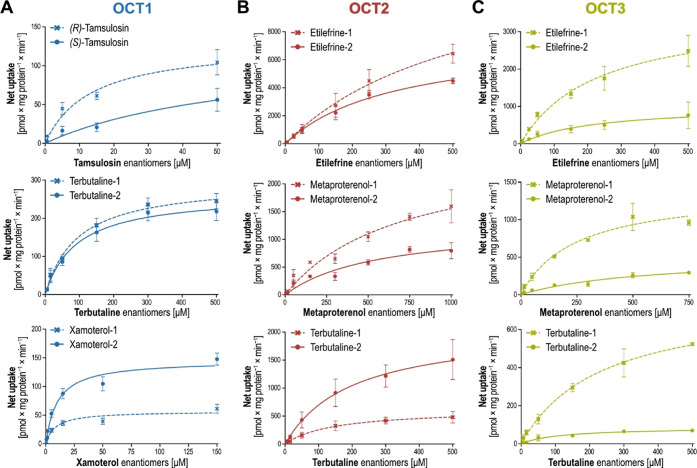
Stereoselective uptake of (anti)adrenergic drugs. OCT1 (A), OCT2 (B), and OCT3 (C) overexpressing HEK293 cells were incubated for 2 min with increasing concentrations of racemic adrenergic agonists and antagonists. Shown is the net uptake after subtracting the uptake into empty-vector-transfected control cells as the mean ± SEM of three independent experiments.

For the newly tested (anti)adrenergic drugs, OCT1 showed only stereoselective uptake of tamsulosin and xamoterol (Figure 2A). Tamsulosin was transported with a higher apparent affinity for the (*R*)-enantiomer, whereas the transport of xamoterol enantiomers differed in the maximum transport capacities. In contrast, OCT2 and OCT3 stereoselectively transported most adrenergic agonists (Figure 2B,C). The enantiomers of terbutaline differed especially by 3.35- and 14.3-fold in their transport capacities at OCT2 and OCT3, respectively. Interestingly, the stereopreferences of OCT2 and OCT3 were opposite for these enantiomers.

Additionally, OCT2 and OCT3 showed stereoselective uptake of etilefrine and metaproterenol (Figure 2B,C), and OCT2 also mediated selective transport of pirbuterol (Figure S2). Adrenergic receptor antagonists were generally weaker OCT substrates compared to beta-adrenergic agonists. Concerning stereoselectivity, OCT2 showed moderate selectivity in the uptake of sotalol enantiomers (Figure S2).

**Figure 3:**
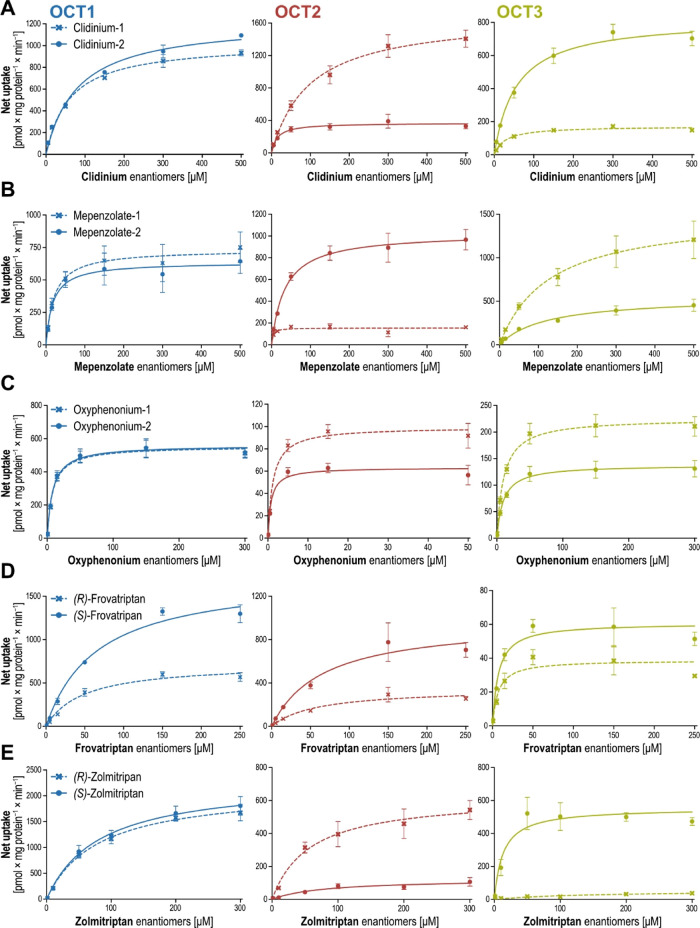
OCT transport kinetics of muscarinergic acetylcholine receptor antagonists and triptans. HEK293 cells overexpressing OCT1, OCT2, and OCT3, and empty-vector (EV control)-transfected control cells were incubated with racemic clidinium (A), mepenzolate (B), oxyphenonium (C), frovatriptan (D), or zolmitriptan enantiomers (E) for 2 min. The net uptake is shown after subtracting the uptake into empty-vector-transfected control cells. Data is presented as mean ± SEM of three independent experiments.

Several muscarinic acetylcholine receptor antagonists belong to the best OCT substrates, which is supported by their overall high intrinsic clearances for all three of the OCTs (Table S1). Interestingly, clidinium and mepenzolate showed highly stereoselective uptake by OCT2 and OCT3 but with opposite stereopreferences (Figure 3A+B).

Also, oxyphenonium was transported with moderate selectivity by OCT2 and OCT3 (Figure 3C). OCT1 showed minor but statistically significant stereoselective uptake of clidinium enantiomers but no selective uptake for mepenzolate and oxyphenonium. Aclidinium is used as a long-acting inhaled anticholinergic agent in obstructive pulmonary disease. Its cell uptake was mediated both by OCT1 and by OCT2, and both transporters favored the transport of (*S*)-aclidinium over the (*R*)-enantiomer (Figures S1 and S2). In contrast, homatropine was an exclusive OCT2 substrate (Table S1). The homatropine enantiomers had a similar *v*_max_ but moderate differences in apparent affinity (Figure S2).

Frovatriptan uptake was characterized by a stereoselective transport by all OCTs with *v*_max_ ratios of 2.39, 2.79, and 1.56 in favor of (*S*)-frovatriptan for OCT1, OCT2, and OCT3, respectively (Figure 3D). Interestingly, the structurally related zolmitriptan was taken up with remarkably high and opposite stereoselectivity by OCT2 and OCT3. OCT2 showed a preference for (*R*)-zolmitriptan, while OCT3 almost exclusively transported only the (*S*)-enantiomer (Figure 3E). Whereas in our study stereoselectivity mostly affected the maximum transport velocity *v*_max_ in most substrate–transporter combinations, OCT3 uptake of zolmitriptan showed high selectivity ratios in both, the concentration at half-maximum transport activity (*K*_m_) and maximum transport activity (*v*_max_), although the Km ratio did not reach statistical significance. This resulted in even higher stereoselectivity ratios for the intrinsic clearances of about 112-fold for (*S*)-zolmitriptan. Ethambutol is an OCT substrate lacking any ring system in its structure. Although it has two chiral centers, due to an internal plane of symmetry, only three instead of four stereoisomers exist. However, the three stereoisomers showed similar uptake by OCTs. All OCTs showed slightly higher maximum uptake velocity of (*R*,*S*)-ethambutol compared to that of the enantiomeric pair (Table S1). The antipsychotic drug amisulpride was transported only by OCT1 but without any stereoselectivity (Figure S1 and Table S1). Milnacipran, a serotonin–norepinephrine reuptake inhibitor, showed half-maximum transport activity by OCT1 at low concentrations of OCT1 but no effects of stereoselectivity. However, OCT2 and OCT3 displayed minor stereoselectivity in milnacipran transport (Figures S2 and S3 and Table S1).

**Figure 4:**
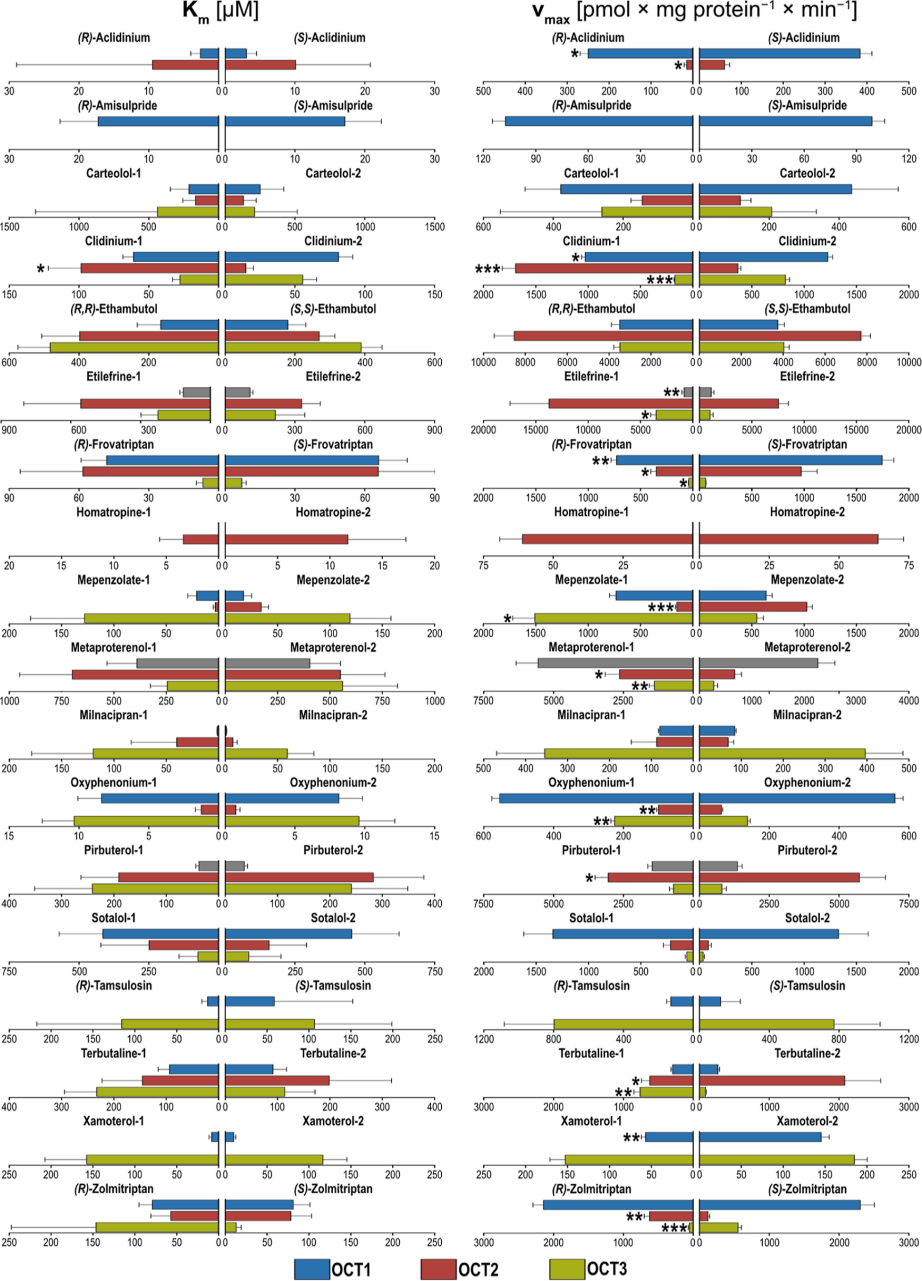
Stereoselectivities in *K*_m_ and *v*_max_ of chiral OCT substrates. Asterisks indicate statistical significance of the differences between the two enantiomers (Student’s *t*-test; *p < 0.05, **p < 0.01, ***p < 0.001). Missing bars indicate no net transport and accordingly also no transport kinetic constants could be identified. Etilefrine, metaproterenol, and pirbuterol were not tested for OCT1 in this study and shown in gray as those were investigated previously.^28^

Generally, in the OCT uptake, maximum transport velocity *v*_max_ was more frequently stereoselective than *K*_m_ (Figure 4). All in all, OCT1, −2, and −3 showed statistically significant differences in *v*_max_ for 3 out of 14 (21.4%), 8 of 15 (53.3%), and 8 of 15 (53.3) investigated substances, respectively. A commonly observed pattern of *v*_max_ stereoselectivity is illustrated by clidinium, mepenzolate, terbutaline, and zolmitriptan. In all cases, OCT1 uptake was nonselective, whereas OCT2 and OCT3 showed high and opposite stereoselectivity. In contrast, only clidinium transport via OCT2 was characterized by statistically significant differences in affinities or concentration at half-maximum transport activity (*K*_m_).

### Influence of OCT1 and OCT2 Genetic Polymorphisms on Stereoselectivity

In most human populations, the genes of OCT1 and OCT2 carry many inherited polymorphisms. However, the activity of only OCT1 is highly variable in most human populations due to frequent polymorphisms with decreased or lost function. Many polymorphisms of OCT2 have no major functional implications except for the Alanine270Serine variant. To test whether their most common polymorphisms affect transporters’ stereoselectivity, we investigated the uptake of the studied substances at single concentrations for the OCT1_Met420del, OCT1_Arg61Cys, and OCT2_Ala270Ser variants. Based on the uptake kinetics for the wild-type transporter, we used a single substrate concentration of either 10, 100, or 1000 μM.

**Figure 5:**
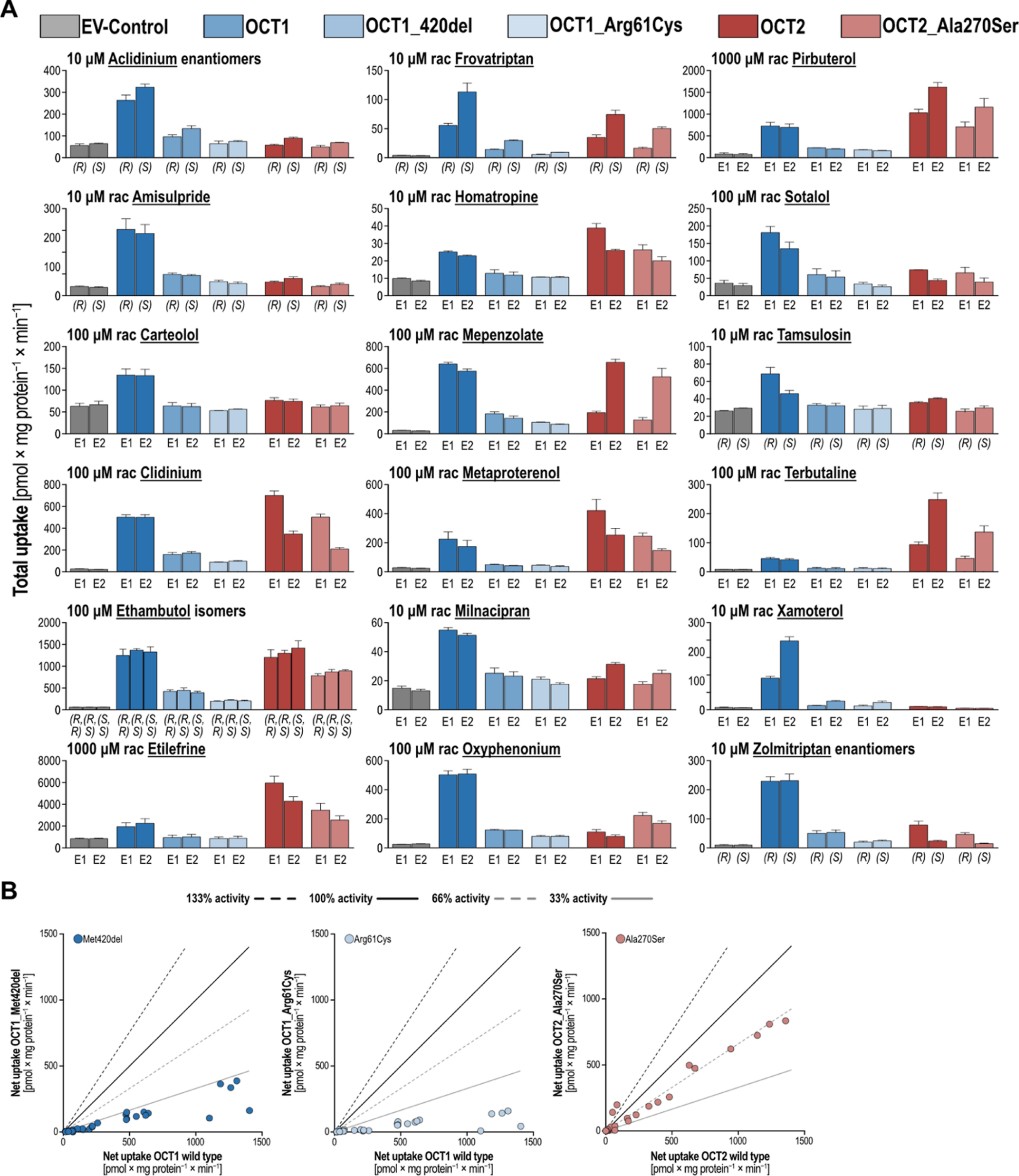
Influence of common functional polymorphism of OCT1 and OCT2. HEK293 cells overexpressing OCT1, OCT2, and empty-vector (EV-)-transfected control cells were incubated with either 10, 100, or 1000 μM substrate for 2 min (A). Intracellular concentrations were quantified by liquid chromatography–tandem mass spectrometry (LC–MS/MS) analysis and are represented by total uptake data with mean ± SEM of three independent experiments. Influence of transporter polymorphisms on the overall transporter activity (B).

Although the genetic polymorphisms had a significant impact on overall uptake rates (Figure 5B), no major changes in the stereoselectivity were observed. Especially the two OCT1 polymorphisms severely impaired substrate uptake leading to uptake rates below 33% of wild-type OCT1. This low activity in the selection of chiral substances studied here was surprising considering that particularly the functional effects of the OCT1 methionine 420 deletion are substrate-dependent and had almost normal activity with several other substrates.^12^ In contrast to those, the Ala270Ser polymorphisms of OCT2 had only a moderate effect on transporter function with activities of around 66% of the wild-type activity. Oxyphenonium enantiomers were the only substrates showing higher uptake by the Ala270Ser variant of OCT2 compared with the wild type.

### Combined Stereoselectivities of OCT1 and CYP2D6 on the Cellular Disposition of Shared Substrates

Stereoselectivity in hepatic biotransformation might be the product of stereoselectivity at an uptake transporter and an intracellularly localized enzyme. To investigate combined effects of OCT1 and CYP2D6 on chiral substrates, we analyzed the cellular disposition of racemic formoterol, oxyphenonium, and tamsulosin in single- and double-transfected cells. Formoterol was shown previously to be stereoselectively transported by OCT1,^28^ whereas we observed stereoselectivity in tamsulosin but not oxyphenonium uptake in this study.

**Figure 6:**
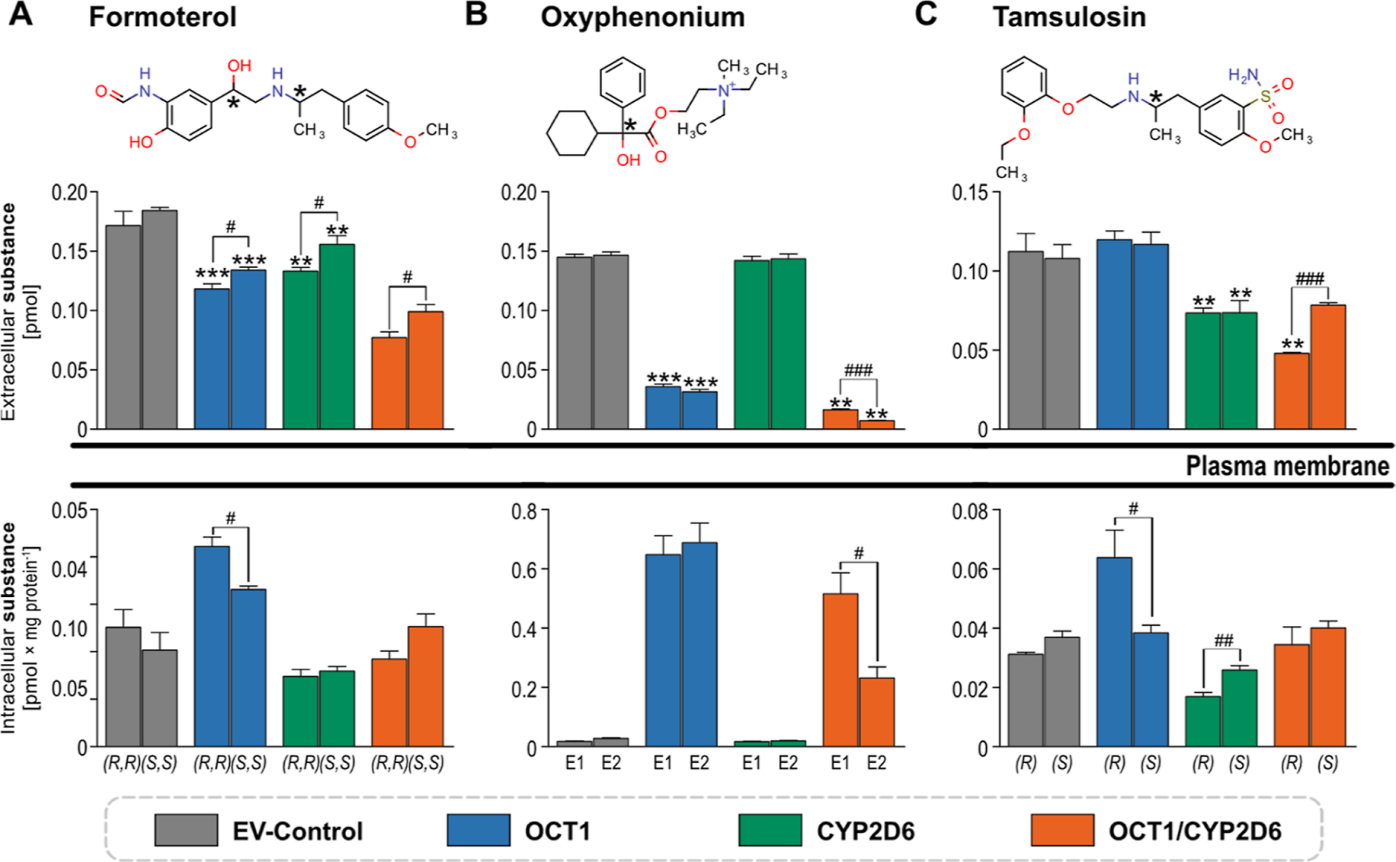
Combined effects of OCT1 and CYP2D6 on the cellular disposition of racemic drugs. HEK293 cells overexpressing OCT1, CYP2D6, and both, and empty-vector (EV)-transfected control cells were incubated with 1 μM racemic formoterol (A), oxyphenonium (B), or tamsulosin (C). After 90 min, the amount of the substance left over in the cellular supernatant as well as the intracellular concentrations were quantified by chiral LC–MS/MS analysis. Results are shown as mean ± SEM of three independent experiments. Chiral centers of OCT1/CYP2D6 substrates are highlighted with asterisks. Asterisks indicate statistical significance of contributions of OCT1, CYP2D6, or the combination of OCT1 and CYP2D6 in the double-transfected cells to the depletion of the individual enantiomer, *p < 0.05, **p < 0.01, ***p < 0.001. The number signs indicate statistically significant differences between two enantiomers with Student’s *t*-test and ^#^*p* < 0.05, ^##^*p* < 0.01, ^###^*p* < 0.001.

Formoterol showed additive effects not only on its cellular disposition but also on its stereoselectivity. OCT1 and CYP2D6 had a preference for the (*R*,*R*)-enantiomer over the corresponding (*S*,*S*)-enantiomer (Figure 6A). OCT1 uptake of oxyphenonium is not stereoselective. However, the double-transfected cells revealed the stereoselective metabolism of oxyphenonium by CYP2D6 (Figure 6B). Single CYP2D6-transfected cells show no metabolism at all due to poor membrane diffusion of oxyphenonium in the absence of carrier-facilitated uptake. Substrate depletion of tamsulosin in the cellular supernatant was primarily mediated by CYP2D6 as the effect of OCT1 did not exceed the EV control (Figure 6C). However, a cooperative effect of the OCT1 and CYP2D6 was observed in the double-transfected cells but only for the depletion of the (*R*)-enantiomer of tamsulosin. Analysis of intracellular tamsulosin concentrations revealed a moderate uptake via OCT1 with a stereopreference for (*R*)-tamsulosin. Interestingly, CYP2D6 showed similar stereoselectivity and accordingly a higher metabolism of (*R*)-tamsulosin. Both effects contribute to the stronger extracellular substrate depletion of (*R*)-tamsulosin.

## Discussion and Conclusions

In this study, we provide a comprehensive characterization of the stereoselectivity in transport by SLC22 organic cation transporters. We tested numerous drugs from different therapeutic areas, and with β-adrenergic agonists and muscarinic acetylcholine receptor antagonists, also several of the best OCT substrates. The most prominent finding was that only OCT2 and OCT3 showed high stereoselective uptake of several substrates. Generally, their stereoselectivity is much higher than that of OCT1, which is in accordance with previous reports.^26,28^ With none of the substrates, there was absolutely zero transport for one of the enantiomers and a relevant transport for the other enantiomer, but as illustrated in Figures 2 and 3, particularly terbutaline and zolmitriptan transport by OCT3 was highly enantioselective.

**Figure 7:**
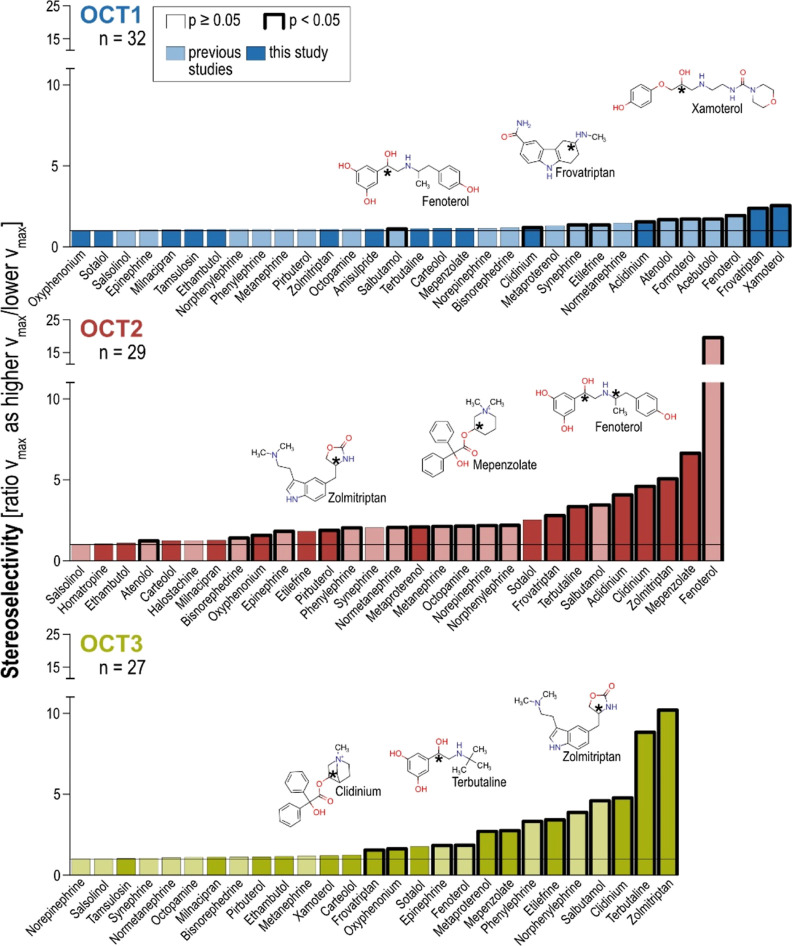
Overview of stereoselectivity of SLC22 organic cation transporters. *V*_max_ ratios are shown for all OCT substrates in ascending order where data on stereoselective transport kinetics were available.^26,28^ Data are presented as the *v*_max_ ratio of the higher transported enantiomer over the other. Substrates investigated in this study are highlighted by dark filling, whereas data taken from the literature are indicated by lighter fillings. The thickness of the borders indicates whereas stereoselectivity ratios were significant according to Student’s *t*-test. The vertical lines represent a ratio of 1 and by this no stereoselectivity. The three most selectively transported substrates are shown with structures where chiral centers are indicated by asterisks.

The integrative overview of available data on stereoselective OCT transport (Figure 7) confirms that highly stereoselective OCT1 transport is an exception. Not only is any significant stereoselectivity much less frequently compared to the other OCTs (34% versus 68 and 48% for OCT2 and OCT3, respectively) but also with the highest OCT1 enantiomeric ratio of 2.56 for xamoterol, the extent of stereoselectivity is remarkably lower compared with some chiral OCT2 or OCT3 substrates. Interestingly, most stereoselectively transported OCT1 substrates have more than one aromatic ring. However, a systematic analysis of how basic chemical properties of OCT substrates might influence stereoselectivity did not reveal any finally conclusive correlations (Figure S4). Regarding the number of rings, the group sizes were too small for reliable conclusions.

Compared with OCT1, most of the OCT2 substrates were taken up stereoselectively, and only the minority of the OCT2 substrates showed unselective transport. Additionally, OCT2 showed the highest selectivity with fenoterol and a corresponding ratio of 25. This is likely not relevant for clinical pharmacokinetics of racemic fenoterol since only a minor fraction of fenoterol is eliminated unchanged via the kidneys. However, it may play a role in some effects of fenoterol on the kidneys. Most importantly, it illustrates that the OCT2 can indeed transport with a pretty high stereoselectivity. Moreover, several other substrates are transported with high selectivity ratios greater than 3. OCT3 is characterized by a more differential distribution of the stereoselectivity in its transport. Numerous substrates are transported highly stereoselectively, whereas half of the investigated substrates showed no selectivity. These differences in stereoselectivity may indicate that OCT1 has more flexible substrate-binding sites compared with those of OCT2 and OCT3. Alternatively, the substrate translocation or the substrate release from the inward-open configuration of the transporter may be more specific in OCT2 and OCT3 compared with OCT1. Ethambutol is the only purely aliphatic chiral OCT substrate studied thus far concerning stereoselective transport. Because of its high flexibility, it may fit in several configurations into the organic cation transporters, explaining the low stereoselectivity of ethambutol transport.

Another interesting finding was that OCT2 and OCT3 frequently showed opposite stereoselectivities. This appears surprising given their close relationship with 50% amino acid identity and 69% amino acid similarity.^6^ However, mutagenesis studies of OCT1 demonstrated that single amino acids can completely determine a transporters’ stereoselectivity.^27^ Although this has not been shown for OCT2 and OCT3 yet, the revolution of structural biology by the improvements in cryogenic electron microscopy (cryo-EM) has just affected the OCT field.^17,29^ High-resolution structural data might accelerate the identification of amino acids determining the stereoselectivity of OCT2 and OCT3 by computationally guided biochemical studies. The frequent naturally occurring genetic variants studied here did not affect the transporters’ stereoselectivity. Nevertheless, as shown previously for OCT1,^27^ single amino acid substitutions can cause completely reversed stereoselectivity. Taking this into consideration, the effects of other naturally occurring OCT1 polymorphisms might be interesting to study. Several of these had no functional consequences but that was only assessed based on a limited number of substrates and without taking stereochemistry into account.^12^

All prominent differences in transport kinetics identified here were differences in the maximum transport rate (Figures 2, 3, and 4 and Table S1). Although with the recent cryo-EM data,^17,29,30^ we have much better structural insights into the OCTs than before, we still do not understand all aspects of the transport processes. We may speculate that the maximum transport rate is not (or not only) correlated with a high binding affinity to the transporter in the outward-open configuration but with rapid substrate release from the inward-open configuration. Thus, for a better understanding of the enantiospecific differences of OCT2-mediated transport of fenoterol or the OCT3-mediated transport of terbutaline or zolmitriptan, we would need further experimental cryo-EM data with the enantiomers, and we would expect that there are differences in the affinity of the enantiomers to the protein in that configuration.^30^ In addition, there may be substrate-dependent differences in the translocation process. Although the enantiomeric differences with fenoterol, terbutaline, and zolmitriptan were highly significant and around 10-fold, it is questionable whether, with these moderate differences, different binding in cryo-EM could be demonstrated. Therefore, for a better understanding, the most realistic next step might be further site-directed mutagenesis studies, which can now be guided by the existing protein structural data.

Concerning clinical relevance, drug transporters are not an isolated system but part of a whole network of proteins involved in the handling of drugs. Studies with coexpression of OCT1 and CYP2D6 illustrated that stereoselectivities of different processes might behave in an additive manner as shown for formoterol and tamsulosin. More interestingly, CYP2D6 displayed a highly stereoselective metabolism of oxyphenonium. However, this was not detectable in single-transfected cells (Figure 6). Only the OCT1-mediated intracellular accumulation of oxyphenonium in the double-transfected cells facilitated efficient drug metabolism and thereby revealed CYP2D6 stereoselectivity. In combination with the known high stereoselectivity in drug metabolism by CYP2D6 and other enzymes,^31^ also minor effects of stereoselectivity in the transport might be relevant for chiral pharmacokinetics. Also, not only CYP enzymes but also other drug-metabolizing enzymes have been described to be stereoselective. For instance, some human phenol sulfotransferases (PSTs) have been described to mediate sulfate conjugation of fenoterol in a highly stereoselective manner with a preference for its (*R*,*R*)-enantiomer.^32^ With the same stereoselectivity of OCT1 for fenoterol^28^ and the in vivo demonstrated relevance of OCT1 for fenoterol pharmacokinetics,^13^ OCT1 and PSTs might control hepatic fenoterol handling in a stereoselective manner.

Concerning clinical pharmacokinetics and therapeutic effects or adverse effects, the differences revealed by our experiments may not be relevant. All of the most prominent differences between the enantiomers were differences in *V*_max_. However, *V*_max_ is relevant at concentrations much higher than the typical blood concentrations of the drugs studied here (most of them are highly potent drugs). Also, the locally higher concentrations, for instance in the portal vein blood relevant for OCT1 and OCT3 in the hepatocytes, are still significantly lower than the *K*_m_ estimates of our study (Figures 2 and 3 and Table S1). But that may be different in less-potent drugs used in higher doses. In general, enantiomeric differences at OCT1 and at OCT3 can be relevant for hepatic uptake and the differences at OCT3 also for effects and adverse effects in the CNS and several other organs. In contrast, enantiomeric differences at the OCT2 may be most relevant for tubular secretion.

Ethambutol is a drug used with single doses of up to 4 g in which *v*_max_ differences would matter if there were such differences. Ethambutol was already earlier identified as a substrate of OCT1, OCT2, OCTN1, and OCTN2,^33^ but here we were interested in the enantiospecific differences because the enantiomers differ in their effects and in their ocular toxicity. Ethambutol is mostly eliminated via the kidneys and renal impairment is associated with increased risk of optic neuropathy.^34^ Thus, interactions at OCT2 as the major transporter of ethambutol may increase the risk of optic neuropathy. Clinically, nowadays, the pure (*S*,*S*)-enantiomer is used in the treatment of tuberculosis. From the clinically used 5-HT_1B/1D_ receptor agonists, about 50% are achiral (e.g., sumatriptan, almotriptan, naratriptan, and rizatriptan) and others are chiral and used as pure enantiomers. Eletriptan is clinically used as the (*R*)-enantiomer, but because of its high lipophilicity, its cell uptake is not relevantly enhanced by the OCTs.^23^ Frovatriptan is clinically used as the pure (*R*)-enantiomer and zolmitriptan as the pure (*S*)-enantiomer. Although the clinically used frovatriptan (*R*)-enantiomer is less dependent on the OCTs, the three transporters may still have some pharmacokinetic relevance (Figure 4). Zolmitriptan is used as the pure (*S*)-enantiomer, which is extensively transported by OCT3 (Figure 4). Thus, for instance, interactions at OCT3 or the rare genetic polymorphisms of OCT3^17^ may have clinical relevance in certain drug combinations or in carriers of the more rare OCT3 genetic polymorphisms. Reasons for marketing (*R*)-frovatriptan and (*S*)-zolmitriptan are not fully disclosed, but (*S*)-zolmitriptan may have a moderately higher 5HT1D receptor binding than the (*R*)-enantiomer.^35^ Numerous anticholinergic drugs are substrates of OCT1, −2, and −3. Therapeutically, they are used as antispasmodic agents and as antisecretory agents in obstructive lung diseases and in urology. Clidinium, mepenzolate, and oxyphenonium, showing substantial steric differences in transport (Figure 3), were earlier considered as gastric acid-reducing drugs, which is irrelevant nowadays, but for other indications, the differences may still be relevant.

Altogether, with some substrate–transporter combinations, OCTs were capable of highly stereoselective substrate translocation, which was not expected considering the pronounced polyspecificity of OCTs.^36^ Nevertheless, our study showed that there can be remarkable differences in stereospecificity between the three closely related transporters, and the stereospecificity of OCT2 and OCT3 appeared to be much more stereospecific than OCT1.

## Experimental Section

### Test Compounds

Compounds were purchased from Santa-Cruz Biotechnology (Darmstadt, Germany; sc-294579, Etilefrine hydrochloride; sc-295159, Homatropine hydrochloride; sc-204086, Milnacipran hydrochloride; and sc-203699, Sotalol hydrochloride), Sigma-Aldrich (Darmstadt, Germany; SML2868, aclidinium bromide; A2729, amisulpride; B8684, bambuterol hydrochloride; B5274, bupivacaine hydrochloride; BP567; carteolol hydrochloride; 492051, choline chloride–trimethyl-*d*_9_; C0414, clidinium bromide; E4630, (*S*,*S*)-ethambutol dihydrochloride; PHR2703, formoterol fumarate; 1286402, frovatriptan; 1286413, (*R*)-frovatriptan succinate; M2398, metaproterenol hemisulfate; M5651, mepenzolate bromide; L5783, *N*-ethyl lidocaine bromide; O5501, oxyphenonium bromide; 32142, pirbuterol acetate; G7048; proguanil hydrochloride; Y0000653, tamsulosin; T1330, (*R*)-tamsulosin hydrochloride; T2528, terbutaline hemisulfate; Y0001986, (*R*)-zolmitriptan; and SML0248, (*S*)-zolmitriptan) and Toronto Research Chemicals (Toronto, Canada; A190150, (*S*)-aclidinium bromide; A633255, (*R*)-amisulpride; E889805, (*R*,*R*)-ethambutol dihydrochloride; E67805, rel-(*R*,*S*)-ethambutol dihydrochloride; and X499808; Xamoterol hemifumarate). Those substances without assigned chirality are racemic mixtures. All compounds had purities of at least 95% according to their manufacturers, as determined by high-performance liquid chromatography (HPLC) analysis. We have reanalyzed representative compounds regarding purity by UV-HPLC (Figure S5) and enantiomeric purity of racemic drugs by chiral HPLC (Figure S6).

### Cellular Uptake and Metabolism Experiments

All transport and metabolism experiments were carried out in stably transfected HEK293 cells. OCT1, OCT2 (wild-type as well as the OCT1-420del, OCT1-R61C, and OCT2-A270S variants) cells, and CYP2D6-single and OCT1/CYP2D6-overexpressing cells were generated using the Flp-In system (Thermo Fisher Scientific, Darmstadt, Germany) as described previously.^22,37,38^ OCT3 overexpressing HEK293 cells were a kind gift from Drs Koepsell and Gorbulev (University of Würzburg, Würzburg, Germany). Amino acid sequences of overexpressed OCTs are given in Figure S7. Cells were cultivated for no longer than 30 passages in Dulbecco’s modified Eagle medium (DMEM, pH 7.4) supplemented with 10% (v/v) FCS, penicillin (100 U/mL), and streptomycin (100 μg/mL).

For cellular uptake measurements, 300,000 HEK293 cells were plated 2 days ahead of the experiment in poly-d-lysine-coated 24-well plates. Cells were washed once with 1 mL of prewarmed HBSS+ (Hanks balanced salt solution supplemented with 10 mM HEPES, pH adjusted to 7.4, 37 °C) prior to substrate addition. Then, cells were incubated with the substrate dissolved in prewarmed HBSS+ for 2 min. Transport was stopped by adding ice-cold HBSS +, followed by two washing steps. Subsequently, cells were lysed with 80% (v/v) acetonitrile containing the appropriate internal standard for HPLC-MS/MS analysis. For every experiment, additional wells per cell line were lysed using RIPA buffer (pH 7.4) for eventual protein quantification using a bicinchoninic acid assay.^39^ Uptake data were later normalized to cellular protein content. Absolute drug concentration quantification was done by comparison to a standard curve with known substance concentrations.

For uptake and metabolism experiments, cells were plated as described for the simple uptake experiments. After the initial washing, cells were incubated with 1 μM racemic substrate dissolved in DMEM supplemented with 20 mM HEPES adjusted to pH 7.4 at 37 °C in a humidified atmosphere inside the cell culture incubator for 90 min. After this, the cellular supernatant was collected and centrifuged at low speed to remove any detached cells. The clear supernatant was precipitated using acetonitrile/methanol (ratio 9:1) with an internal standard for subsequent LC–MS/MS analysis.

### Stereoselective Concentration Analyses by LC–MS/MS

Intra- and extracellular substrate concentrations were quantified by HPLC-MS/MS analysis. The Shimadzu Nexera HPLC system was composed of a SIL-30AC autosampler, a CTO-20AC column oven, a LC-30AD pump, and a CBM-20A controller (Shimadzu, Kyoto, Japan). Chiral separation was done on either a CHIRALPAK AGP HPLC column (100 × 2.1 mm inner dimensions, 5 μm particle size; Sigma-Aldrich), a CHIRALPAK CBH HPLC column (100 × 3 mm inner dimensions, 5 μm particle size; Sigma-Aldrich), or an Astec chirobiotic T column (150 × 2.1 mm, 5 μm particle size; Sigma-Aldrich) with the corresponding guard columns. Chromatography was carried out with an aqueous mobile phase buffered with ammonium acetate and supplemented with 2-propanol or methanol as organic modifiers. Detailed chromatographic conditions are listed in Table S2. Order of enantiomer elution was obtained by injecting enantiopure reference compounds or from the available reference literature.^40^ Whenever the order was unavailable, the enantiomers were termed E1 and E2 with E1 referring to the first eluting enantiomer using the chromatographic conditions described in Table S2. Bambuterol, milnacipran, and proguanil were used as internal standards for substances analyzed on the AGP, CBH, and chirobiotic T columns, respectively.

For achiral substance separation, a Brownlee SPP RP-Amide column (4.6 × 100 mm inner dimension with 2.7 μm particle size, PerkinElmer, Waltham, MA) with a C18 precolumn used. Reversed-phase chromatography was carried out at 40 °C with an aqueous mobile phase containing 0.1% (v/v) formic acid and an organic additive (acetonitrile/methanol (6:1), both from LGC Standards, Wesel, Germany), ranging from 3% to 35% (v/v). Isocratic elution was achieved at flow rates of 0.3 or 0.4 mL/min. Mobile phase compositions for achiral chromatography are listed in Table S3.

Compounds were detected with an API 4000 tandem mass spectrometer (AB SCIEX, Darmstadt, Germany) operating in multiple reaction monitoring mode. Peak integration and quantification were performed using Analyst software (version 1.6.2, AB SCIEX). A list of MS detection parameters is summarized in Table S4.

### Calculations

Uptake data were generally normalized to total protein content to account for variation in the seeding densities of different cell lines and of the different experiments performed at different days. Transporter-mediated net uptake was determined as the difference between uptake in transporter-overexpressing cells and the uptake into empty-vector-transfected controls. Net uptake data was fitted by nonlinear regression analysis following the Michaelis–Menten equation (*v* = *v*_max_ × [S]/(*K*_m_ + [S])) using GraphPad Prism (version 5.01 for Windows, GraphPad Software, La Jolla, CA, USA). *V* is the transport velocity, *v*_max_ is the maximum transport velocity, [S] is the substrate concentration, and *K*_m_ is the substrate concentration, which is required to reach half of *v*_max_. The intrinsic clearance Cl_int_ is the ratio of *v*_max_ over *K*_m_.

The depletion of the extracellular substrate and significance of the effects of the OCT1 and CYP2D6 as well as the significance of possible synergistic interactions within the double-transfected cells were analyzed by multiple linear regression analysis following the equation Y = *a* × CYP2D6 + *b* × OCT1 + *c* × CYP2D6/OCT1. *a*, *b*, and *c* are the relative contributions of the factors and Y is the concentration of the substrate after 90 min incubation. CYP2D6/OCT1 denotes the interaction between CYP2D6 and OCT1.

## Supporting Information Available

Net uptake curves of OCT1 for investigated chiral drugs; net uptake curves of OCT2 for investigated chiral drugs; net uptake curves of OCT3 for investigated chiral drugs; correlation of stereoselectivity and basic physicochemical descriptors; representative UV-HPLC traces of investigated drugs; representative chiral HPLC of racemic drugs; amino acid sequences of overexpressed OCTs; kinetic parameters for the stereoselective transport of investigated drugs by OCTs; HPLC conditions for chiral separation of investigated substances; mobile phase compositions of achiral substance separation; and MS detection parameters (PDF)
Molecular formula strings (CSV)

## Abbreviations used

ACh: acetylcholine
AGP: α1-glycoprotein
CBH: cellobiohydrolase
cryo-EM: cryogenic electron microscopy
CYP2D6: cytochrome P450 2D6
HBSS: hanks balanced salt solution
IPA: isopropyl alcohol
LC-MS/MS: liquid chromatography coupled to mass spectrometry
NH_4_Ac: ammonium acetate
OCT: organic cation transporter
SEM: standard error of the mean
