# Supplementary material for: Stereoselectivity in Cell Uptake by SLC22 Organic Cation Transporters 1, 2, and 3
Source: J Med Chem. 2023 Dec 5;66(23):15990–6001. doi: 10.1021/acs.jmedchem.3c01436 (PMC10726348; doi:10.1021/acs.jmedchem.3c01436)
Supplement: Supplementary file 1 — jm3c01436_si_001.pdf [file jm3c01436_si_001.pdf]

Supporting information

Stereoselectivity in cell uptake by SLC22 organic cation transporters 1, 2, and 3

Lukas Gebauer*, Ole Jensen, Muhammad Rafehi, and Jürgen Brockmöller

Institute of Clinical Pharmacology, University Medical Center Göttingen, D-37075 Göttingen, Germany

E-Mail: lukas.gebauer@med.uni-goettingen.de

Table of contents

Figure S1: Net uptake curves of organic cation transporter 1 (OCT1) for investigated chiral drugs	Page S3
Figure S2: Net uptake curves of organic cation transporter 2 (OCT2) for investigated chiral drugs	Page S4
Figure S3: Net uptake curves of organic cation transporter 3 (OCT3) for investigated chiral drugs	Page S5
Figure S4: Correlation of stereoselectivity and basic physicochemical descriptors	Page S6
Figure S5: Representative UV-HPLC traces of investigated drugs	Page S7
Figure S6: Representative chiral HPLC of racemic drugs	Page S8
Figure S7: Amino acid sequences of overexpressed OCTs	Page S9
Table S1: Kinetic parameters for the stereoselective transport of investigated drugs by OCTs	Page S10

Table S2: HPLC conditions for chiral separation of investigated substances Page S14

Table S3: Mobile phase compositions of achiral substance separation Page S15

Table S4: Mass spectrometry detection parameters Page S16

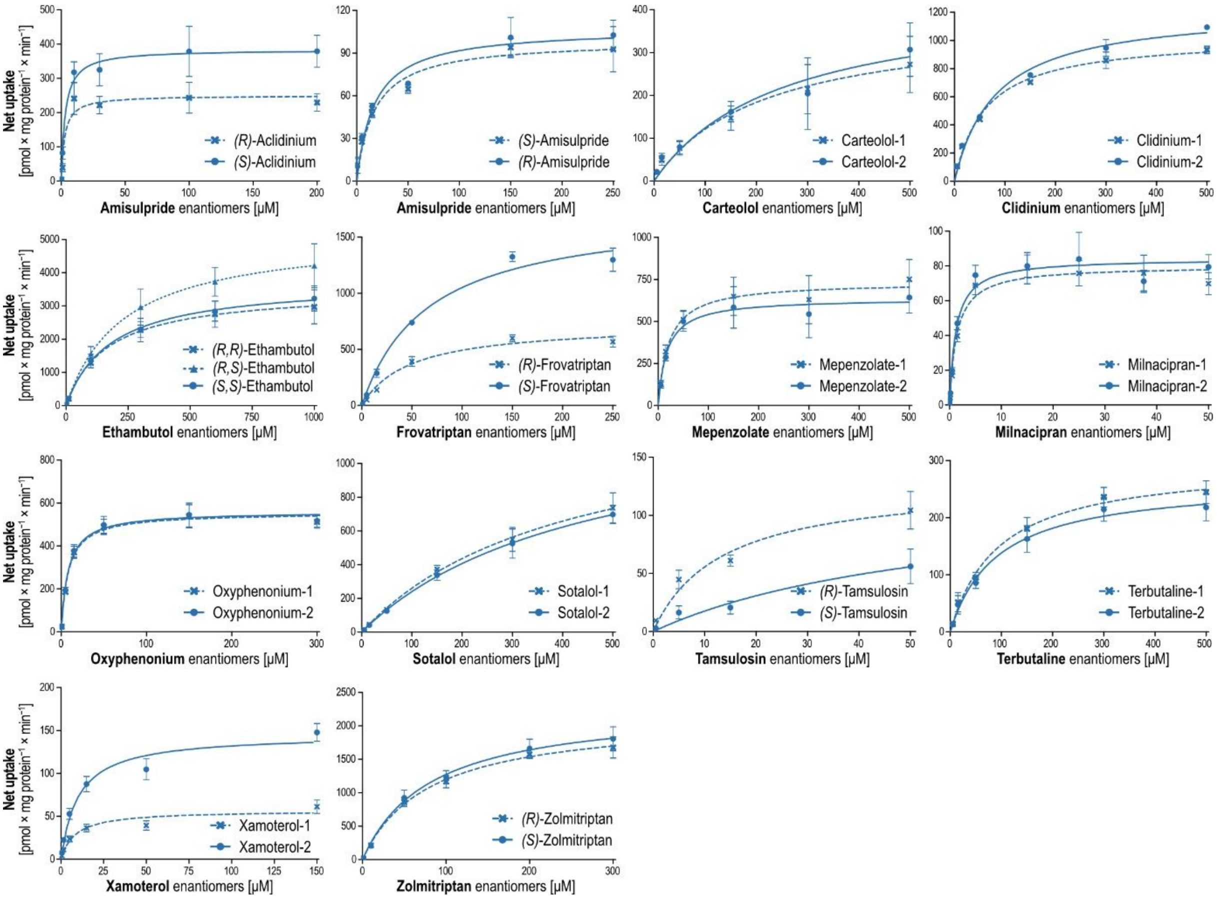

Figure S1: Net uptake curves of organic cation transporter 1 (OCT1) for investigated chiral drugs (in alphabetical order). Data is presented as mean ± SEM of at least three independent experiments. Not transported substances (with no saturable net uptake) are not shown.

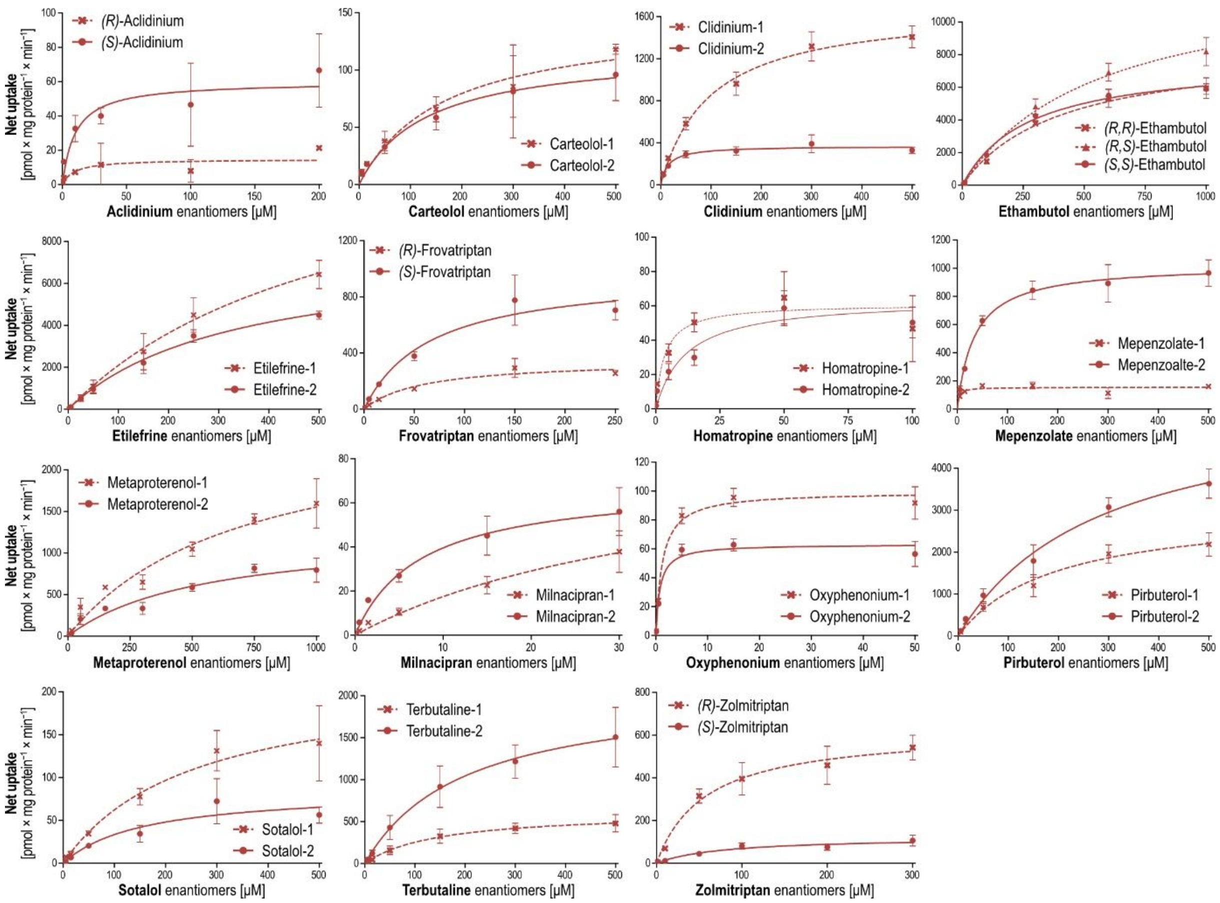

Figure S2: Net uptake curves of organic cation transporter 2 (OCT2) for investigated chiral drugs (in alphabetical order). Data is presented as mean ± SEM of at least three independent experiments. Not transported substances (with no saturable net uptake) are not shown.

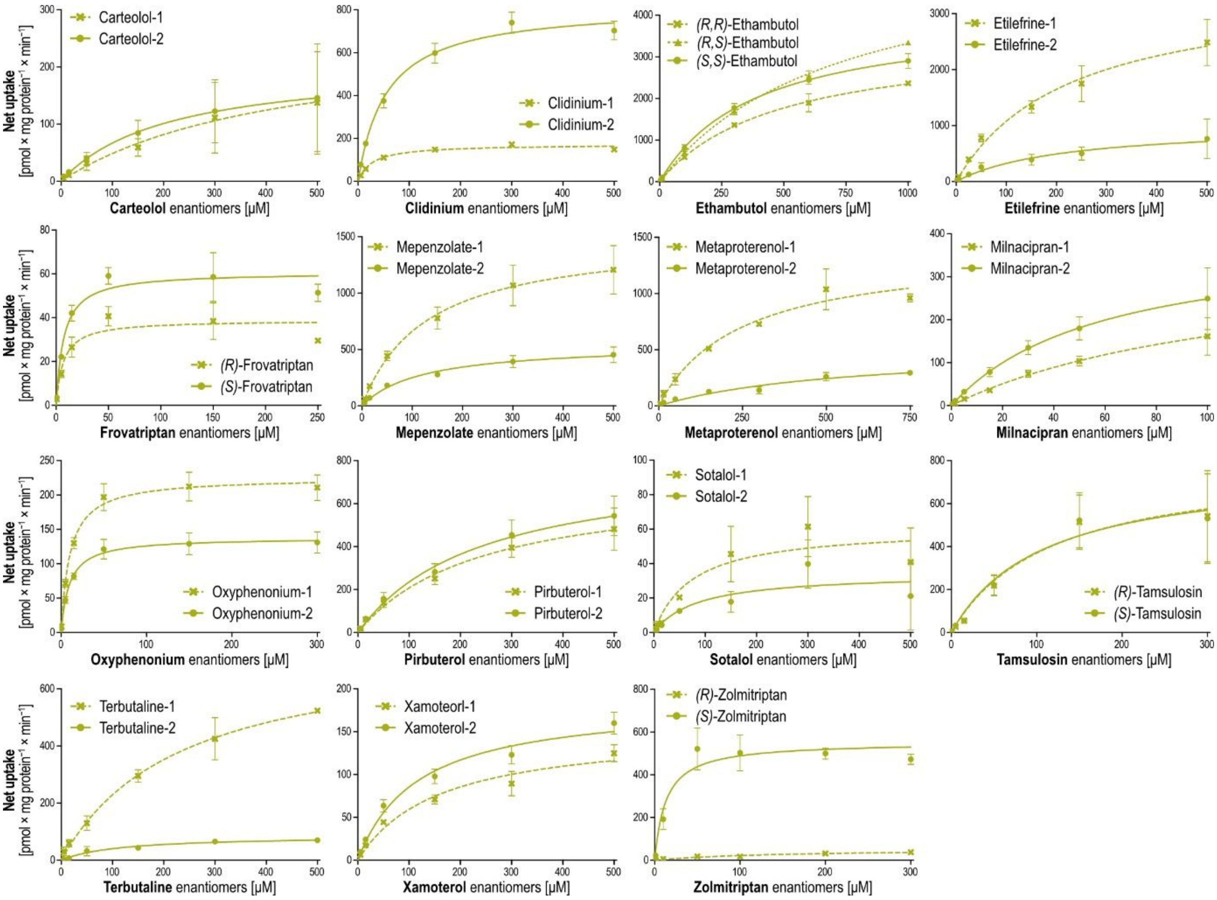

Figure S3: Net uptake curves of organic cation transporter 3 (OCT3) for investigated chiral drugs (in alphabetical order). Data is presented as mean ± SEM of at least three independent experiments. Not transported substances (with no saturable net uptake) are not shown.

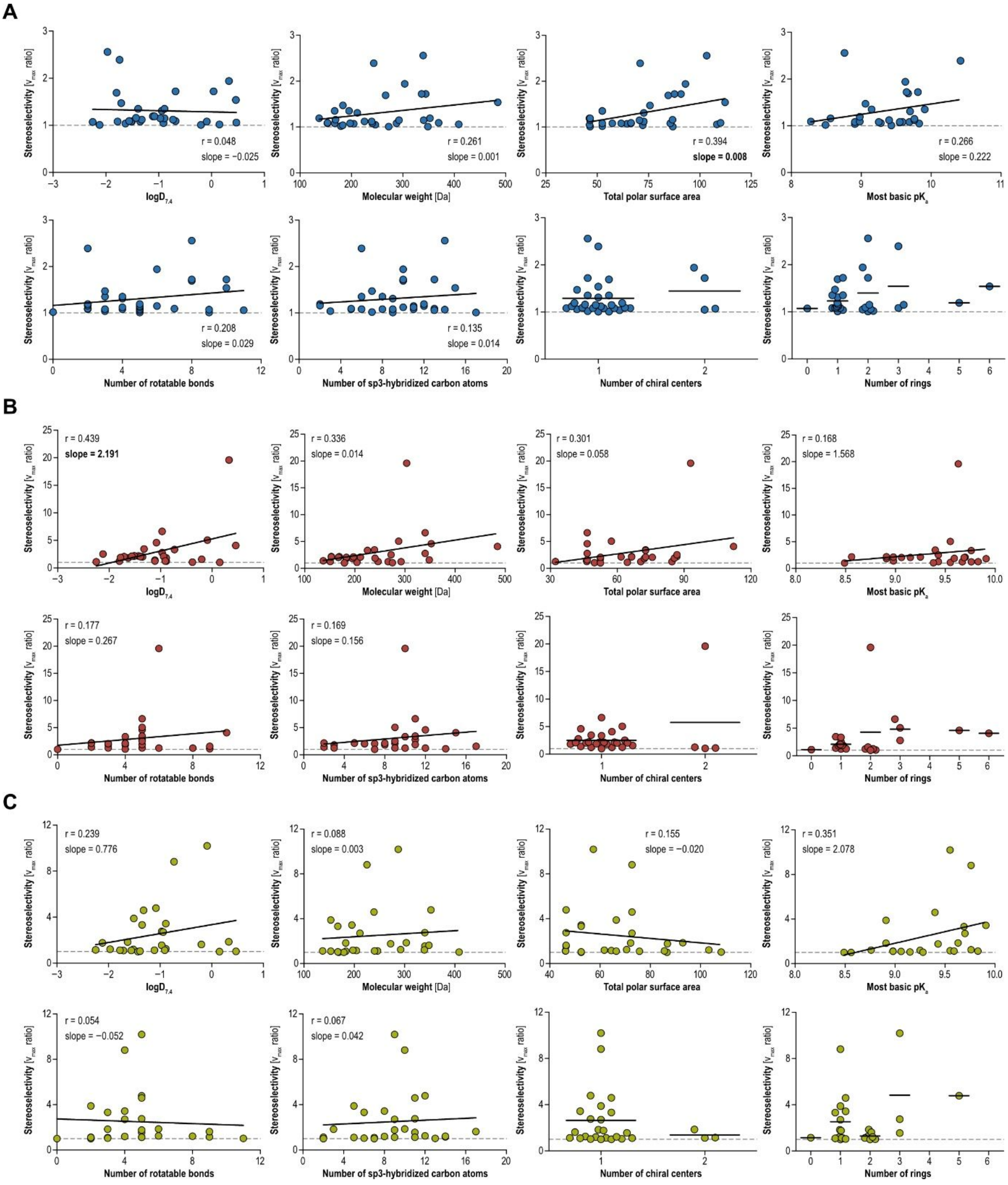

Figure S4: Correlation of stereoselectivity and basic physicochemical descriptors. Stereoselectivity expressed as vmax ratio (vmax of the higher transporter enantiomer over the other) of OCT1 (A), OCT2 (B) and OCT3 (C) related to differend physicochemical properties of the transported substrates.

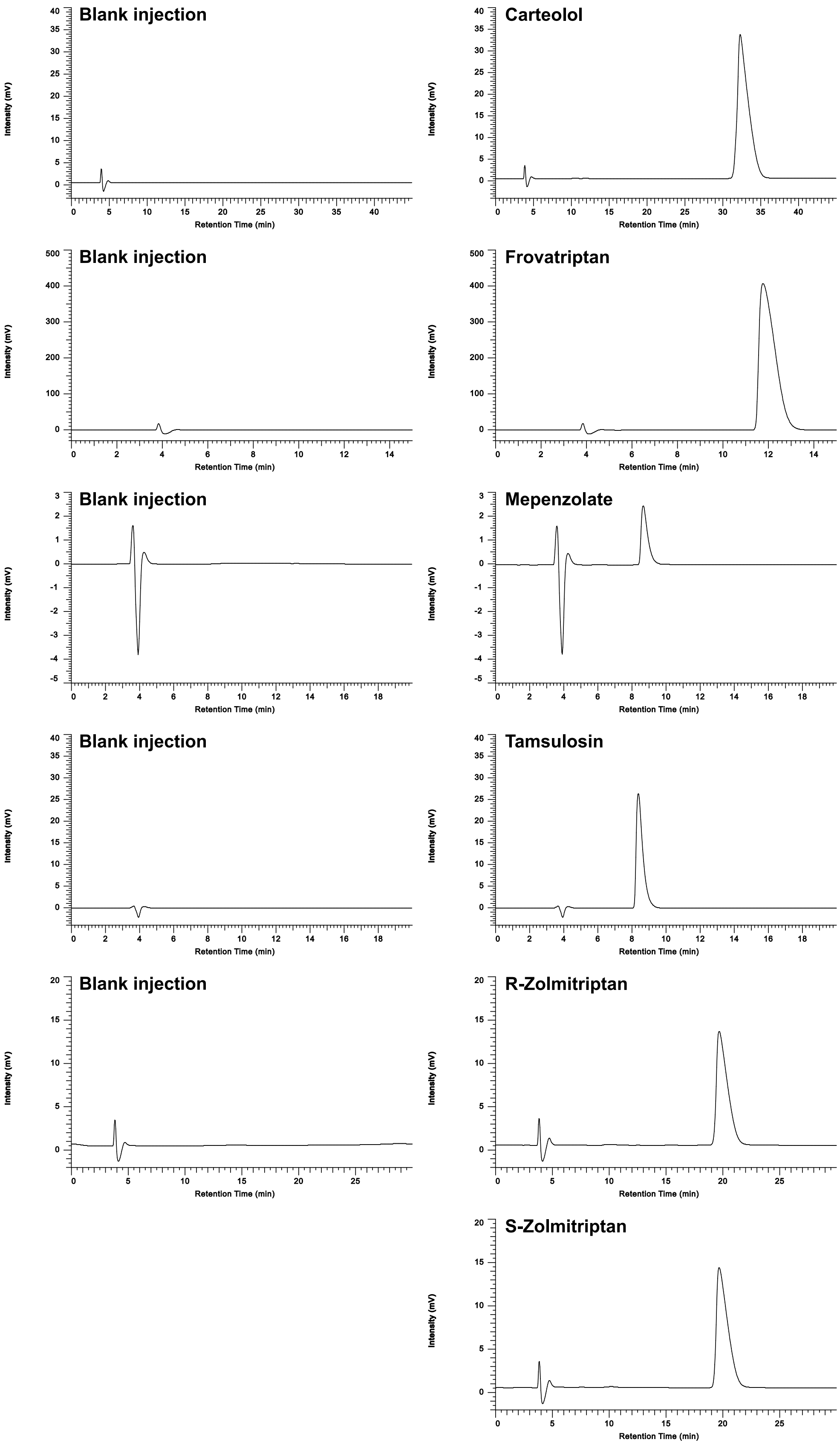

Figure S5: Representative UV-HPLC traces of investigated drugs.

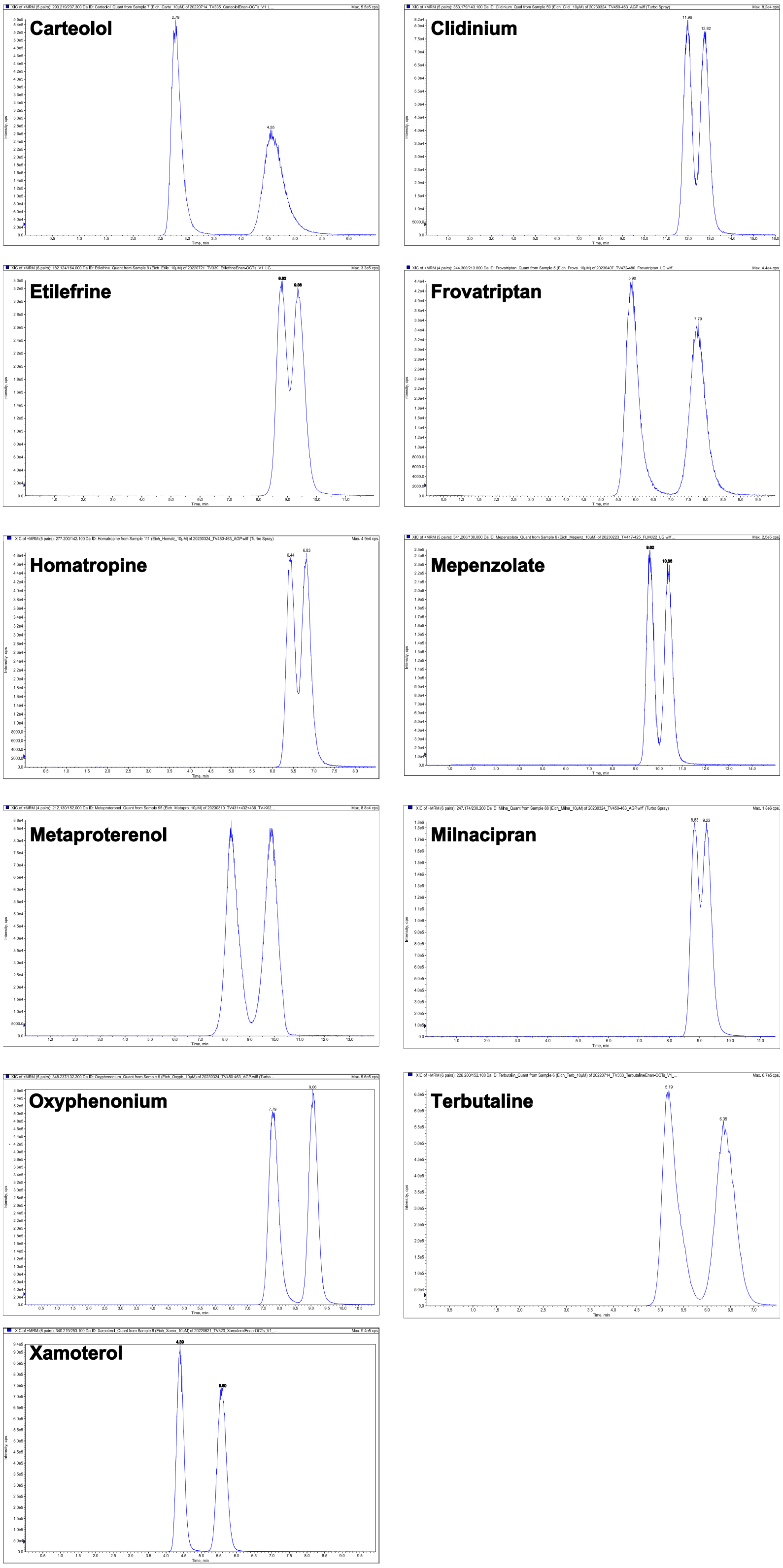

Figure S6: Representative chiral HPLC of racemic drugs. Equal peak areas represent equimolar mixture of individual enantiomers within racemic mixtures of investigated substances.

> Organic cation transporter 1 (OCT1/*SLC22A1*)

```
MPTVDDILEQVGESGWFQKQAFLILCLLSAAFAPICVGIVFLGFTPDHHCQSPGVAELSQRCGWSPAEELNYTVPGLGPAGEA
FLGQCRRYEVDWNQSALSCVDPLASLATNRSHLPLGPCQDGWVYDTPGSSIVTEFNLVCADSWKLDLFQSCLNAGFLFGSLGV
GYFADRFGRKLCLLGTVLVNAVSGVLMAFSPNYMSMLLFRLLQGLVSKGNWMAGYTLITEFVGSGSRRTVAIMYQMAFTVGLV
ALTGLAYALPHWRWLQLAVSLPTFLFLLYYWCVPESPRWLLSQKRNTEAIKIMDHIAQKNGKLPPADLKMLSLEEDVTEKLSP
SFADLFRTPRLRKRTFILMYLWFTDSVLYQGLILHMGATSGNLYLDFLYSALVEIPGAFIALITIDRVGRIYPMAMSNLLAGA
ACLVMIFISPDLHWLNIIIMCVGRMGITIAIQMICLVNAELYPTFVRNLGVMVCSSLCDIGGIITPFIVFRLREVWQALPLIL
FAVLGLLAAGVTLLLPETKGVALPETMKDAENLGRKAKPKENTIYLKVQTSEPSGT
```

> Organic cation transporter 2 (OCT2/*SLC22A2*)

```
MPTTVDDVLEHGGEFHFFQKQMFFLLALLSATFAPIYVGIVFLGFTPDHRCRSPGVAELSLRCGWSPAEELNYTVPGPGPAGE
ASPRQCRRYEVDWNQSTFDCVDPLASLDTNRSRLPLGPCRDGWVYETPGSSIVTEFNLVCANSWMLDLFQSSVNVGFFIGSMS
IGYIADRFGRKLCLLTTVLINAAAGVLMAISPTYTWMLIFRLIQGLVSKAGWLIGYILITEFVGRRYRRTVGIFYQVAYTVGL
LVLAGVAYALPHWRWLQFTVALPNFFFLLYYWCIPESPRWLISQNKNAEAMRIIKHIAKKNGKSLPASLQRLRLEEETGKKLN
PSFLDLVRTPQIRKHTMILMYNWFTSSVLYQGLIMHMGLAGDNIYLDFFYSALVEFPAAFMIILTIDRIGRRYPWAASNMVAG
AACLASVFIPGDLQWLKIIISCLGRMGITMAYEIVCLVNAELYPTFIRNLGVHICSSMCDIGGIITPFLVYRLTNIWLELPLM
VFGVLGLVAGGLVLLLPETKGKALPETIEEAENMQRPRKNKEKMIYLQVQKLDIPLN
```

> Organic cation transporter 3 (OCT1/*SLC22A3*)

```
MPSFDEALQRVGEFGRFQRRVFLLLCLTGVTLAFLFVGVVFLGTQPDHYWCRGPSAAALAERCGWSPEEEWNRTAPASRGPGP
PERRGRCQRYLLEAANDSASATSALSCADPLAAFPNRSAPLVPCRGGWRYAQAHSTIVSEFDLVCVNAWMLDLTQAILNLGFL
TGAFTLGYAADRYGRIVIYLLSCLGVGVTGVVVAFAPNFPVFVIFRFLQGVFGKGTWMTCYVIVTEIVGSKQRRIVGIVIQMF
FTLGIIILPGIAYFIPNWQGIQLAITLPSFLFLLYYWVVPESPRWLITRKKGDKALQILRRIAKCNGKYLSSNYSEITVTDEE
VSNPSFLDLVRTPQMRKCTLILMFAWFTSAVVYQGLVMRLGIIGGNLYIDFFISGVVELPGALLILLTIERLGRRLPFAASNI
VAGVACLVTAFLPEGIAWLRTTVATLGRLGITMAFEIVYLVNSELYPTTLRNFGVSLCSGLCDFGGIIAPFLLFRLAAVWLEL
PLIIFGILASICGGLVMLLPETKGIALPETVDDVEKLGSPHSCKCGRNKKTPVSRSHL
```

Figure S7: Amino acid sequences of overexpressed OCTs.

Table S1 Kinetic parameters for the stereoselective transport of investigated drugs by OCTs

Transporter	Substrate	K_m ± SEM [µM]	V_{max} ± SEM [pmol × mg protein^{-1} × min^{-1}]	Cl_{int} ± SEM [mL × g protein^{-1} × min^{-1}]	Stereoselectivity		
					K_m	V_{max}	Cl_{int}
OCT1	*(R)*-Aclidinium	2.61 ± 1.39	250 ± 19	95.6 ± 58.1	1.17-fold for *(R)*	1.54-fold for *(S)**	1.31-fold for *(S)*
	(S)-Aclidinium	3.06 ± 1.42	384 ± 28	125 ± 67			
	(R)-Amisulpride	17.2 ± 5.2	107 ± 8	6.26 ± 2.35	1.01-fold for *(R)*	1.09-fold for *(R)*	1.09-fold for *(R)*
	(S)-Amisulpride	17.2 ± 5.5	98.9 ± 7.2	5.73 ± 2.24			
	Carteolol-1	212 ± 134	379 ± 102	1.78 ± 1.60	1.18-fold for 1	1.15-fold for 1	1.03-fold for 1
	Carteolol-2	251 ± 169	436 ± 134	1.74 ± 1.70			
	Clidinium-1	61.0 ± 7.6	1028 ± 35	16.9 ± 2.7	1.33-fold for 1	1.19-fold for 2*	1.12-fold for 1
	Clidinium-2	81.1 ± 10.3	1226 ± 46	15.1 ± 2.5			
	(R,R)-Ethambutol	166 ± 67	3492 ± 402	21.0 ± 10.9	1.08-fold for *(R,R)*	1.07-fold for *(S,S)*	1.01-fold for *(R,R)*
	(S,S)-Ethambutol	180 ± 52	3743 ± 317	21.0 ± 7.8			
	(R,S)-Ethambutol	229 ± 80	5175 ± 656	22.6 ± 11.7			
	(R)-Frovatriptan	48.1 ± 11.0	729 ± 52	15.2 ± 4.6	1.37-fold for *(R)*	2.39-fold for *(S)***	1.75-fold for *(S)*
	(S)-Frovatriptan	66.0 ± 12.3	1744 ± 112	26.4 ± 6.6			
	Homatropine-1	*No saturable net uptake*					
	Homatropine-2						
	Mepenzolate-1	20.9 ± 8.5	733 ± 62	35.0 ± 17.2	1.2-fold for 2	1.15-fold for 1	1.04-fold for 2
	Mepenzolate-2	17.5 ± 7.6	637 ± 57	36.3 ± 19.4			
	Milnacipran-1	1.40 ± 0.29	79.9 ± 3.3	57.2 ± 14.2	1.15-fold for 2	1.05-fold for 2	1.21-fold for 2
	Milnacipran-2	1.22 ± 0.24	84.2 ± 3.2	69.3 ± 16.2			
	Oxyphenonium-1	8.39 ± 1.69	554 ± 23	66.1 ± 16.0	1.03-fold for 2	1.01-fold for 2	1.04-fold for 2
	Oxyphenonium-2	8.14 ± 1.71	560 ± 24	68.8 ± 17.4			
	Sotalol-1	415 ± 156	1340 ± 278	3.23 ± 1.89	1.09-fold for 1	1.01-fold for 1	1.10-fold for 1
	Sotalol-2	453 ± 179	1329 ± 283	2.93 ± 1.72			
	(R)-Tamsulosin	13.2 ± 6.9	129 ± 24	9.78 ± 6.97	4.45-fold for *(R)*	1.06-fold for *(R)*	4.74-fold for *(R)*
	(S)-Tamsulosin	58.6 ± 93.8	121 ± 114	2.06 ± 5.24			
	Terbutaline-1	93.9 ± 21.6	297 ± 21	3.16 ± 0.95	1.03-fold for 2	1.12-fold for 1	1.09-fold for 1
	Terbutaline-2	91.3 ± 26.0	264 ± 23	2.90 ± 1.08			
	Xamoterol-1	8.32 ± 3.08	56.8 ± 5.3	6.83 ± 3.16	1.22-fold for 1	2.56-fold for 2**	2.10-fold for 2
	Xamoterol-2	10.1 ± 2.6	145 ± 10	14.4 ± 4.6			

Transporter	Substrate	K_m ± SEM [µM]	V_{max} ± SEM [pmol × mg protein^{-1} × min^{-1}]	Cl_{int} ± SEM [mL × g protein^{-1} × min^{-1}]	Stereoselectivity K_m	Stereoselectivity V_{max}	Stereoselectivity Cl_{int}
**OCT1**	*(R)*-Zolmitriptan	79.0 ± 16.0	2144 ± 151	27.1 ± 7.4	1.03-fold for *(R)*	1.08-fold for *(S)*	1.05-fold for *(S)*
	(S)-Zolmitriptan	81.2 ± 20.1	2305 ± 200	28.4 ± 9.5			
**OCT2**	*(R)*-Aclidinium	9.45 ± 19.5	14.7 ± 6.1	1.56 ± 3.86	1.07-fold for *(R)*	4.07-fold for *(S)**	3.79-fold for *(S)*
	(S)-Aclidinium	10.1 ± 10.7	59.9 ± 12.3	5.91 ± 7.46			
	(R)-Amisulpride	*No saturable net uptake*					
	(S)-Amisulpride						
	Carteolol-1	166 ± 92	145 ± 32	0.88 ± 0.68	1.27-fold for 2	1.24-fold for 1	1.03-fold for 2
	Carteolol-2	131 ± 91	118 ± 30	0.90 ± 0.86			
	Clidinium-1	98.5 ± 23.5	1693 ± 128	17.2 ± 5.4	6.66-fold for 2*	4.60-fold for 1***	1.45-fold for 2
	Clidinium-2	14.9 ± 5.5	368 ± 27	24.9 ± 11.2			
	(R,R)-Ethambutol	398 ± 109	8531 ± 957	21.4 ± 8.3	1.24-fold for *(S,S)*	1.11-fold for *(R,R)*	1.34-fold for *(S,S)*
	(S,S)-Ethambutol	270 ± 45	7718 ± 446	28.6 ± 6.4			
	(R,S)-Ethambutol	559 ± 167	13021 ± 1830	23.3 ± 10.2			
	Etilefrine-1	557 ± 245	13734 ± 3731	24.6 ± 17.5	1.69-fold for 2	1.82-fold for 1	1.07-fold for 1
	Etilefrine-2	329 ± 80	7559 ± 952	23.0 ± 8.5			
	(R)-Frovatriptan	58.3 ± 27.0	349 ± 53	5.98 ± 3.68	1.13-fold for *(R)*	2.79-fold for *(S)**	2.47-fold for *(S)*
	(S)-Frovatriptan	65.8 ± 29.7	972 ± 152	14.8 ± 9.0			
	Homatropine-1	3.37 ± 2.27	61.0 ± 8.1	18.1 ± 14.6	3.47-fold for 1	1.05-fold for 2	3.31-fold for 1
	Homatropine-2	11.7 ± 5.5	64.0 ± 9.2	5.46 ± 3.36			
	Mepenzolate-1	3.19 ± 1.92	155 ± 12	48.5 ± 33.0	10.8-fold for 1	6.64-fold for 2***	1.62-fold for 1
	Mepenzolate-2	17.5 ± 7.7	1026 ± 51	29.9 ± 7.6			
	Metaproterenol-1	699 ± 251	2638 ± 515	3.78 ± 2.10	1.27-fold for 2	2.09-fold for 1*	1.64-fold for 1
	Metaproterenol-2	550 ± 213	1263 ± 243	2.30 ± 1.33			
	Milnacipran-1	40.1 ± 43.4	87.5 ± 60.4	2.18 ± 3.87	5.43-fold for 2	1.27-fold for 1	4.27-fold for 2
	Milnacipran-2	7.38 ± 4.12	68.8 ± 12.8	9.32 ± 6.95			
	Oxyphenonium-1	1.24 ± 0.42	99.5 ± 5.3	80.0 ± 31.0	1.62-fold for 2	1.57-fold for 1**	1.03-fold for 2
	Oxyphenonium-2	0.77 ± 0.30	63.2 ± 3.6	82.4 ± 36.6			
	Pirbuterol-1	191 ± 72	3047 ± 470	15.0 ± 8.5	1.48-fold for 1	1.88-fold for 2*	1.27.fold for 2
	Pirbuterol-2	283 ± 97	5733 ± 932	20.3 ± 10.2			

Transporter	Substrate	K_m ± SEM [µM]	V_{max} ± SEM [pmol × mg protein^{-1} × min^{-1}]	Cl_{int} ± SEM [mL × g protein^{-1} × min^{-1}]	Stereoselectivity K_m	Stereoselectivity V_{max}	Stereoselectivity Cl_{int}
OCT2	Sotalol-1	249 ± 173	217 ± 68	0.87 ± 0.88	1.59-fold for 2	2.53-fold for 1	1.59-fold for 1
	Sotalol-2	157 ± 134	85.7 ± 27.7	0.55 ± 0.64			
	(R)-Tamsulosin	No saturable net uptake					
	(S)-Tamsulosin						
	Terbutaline-1	146 ± 77	622 ± 121	4.27 ± 3.09	1.37-fold for 1	3.35-fold for 2*	2.45-fold for 2
	Terbutaline-2	199 ± 119	2082 ± 516	10.5 ± 8.9			
	Xamoterol-1	No saturable net uptake					
	Xamoterol-2						
	(R)-Zolmitriptan	57.1 ± 23.7	624 ± 78	10.9 ± 5.9	1.37-fold for *(R)*	5.06-fold for *(R)***	6.95-fold for *(R)*
	(S)-Zolmitriptan	78.4 ± 43.1	123 ± 25	1.57 ± 0.81			
OCT3	*(R)*-Aclidinium	No saturable net uptake					
	(S)-Aclidinium						
	(R)-Amisulpride	No saturable net uptake					
	(S)-Amisulpride						
	Carteolol-1	438 ± 874	261 ± 292	0.59 ± 1.85	2.08-fold for 2	1.24-fold for 1	1.66-fold for 2
	Carteolol-2	211 ± 306	207 ± 128	0.98 ± 2.03			
	Clidinium-1	27.7 ± 5.3	172 ± 7	6.22 ± 1.47	2.01-fold for 1	4.78-fold for 2***	2.38-fold for 2
	Clidinium-2	55.5 ± 10.0	822 ± 39	14.8 ± 3.4			
	(R,R)-Ethambutol	483 ± 93	3482 ± 297	7.21 ± 2.00	1.24-fold for *(S,S)*	1.16-fold for *(S,S)*	1.44-fold for *(R,R)*
	(S,S)-Ethambutol	390 ± 60	4035 ± 252	10.4 ± 2.2			
	(R,S)-Ethambutol	725 ± 80	5734 ± 329	7.91 ± 1.32			
	Etilefrine-1	226 ± 73	3510 ± 514	15.6 ± 7.3	1.05-fold for 2	3.43-fold for 1*	3.28-fold for 1
	Etilefrine-2	216 ± 127	1022 ± 282	4.74 ± 4.09			
	(R)-Frovatriptan	6.79 ± 2.75	38.8 ± 3.4	5.72 ± 2.82	1.05-fold for *(R)*	1.56-fold for *(S)**	1.49-fold for *(S)*
	(S)-Frovatriptan	7.13 ± 1.88	60.7 ± 3.5	8.50 ± 2.73			
	Homatropine-1	No saturable net uptake					
	Homatropine-2						
	Mepenzolate-1	128 ± 52	1509 ± 212	11.9 ± 6.4	1.07-fold for 2	2.75-fold for 1*	2.56-fold for 1
	Mepenzolate-2	119 ± 39	549 ± 61	4.61 ± 2.02			

Transporter	Substrate	K_m ± SEM [µM]	V_{max} ± SEM [pmol × mg protein^{-1} × min^{-1}]	Cl_{int} ± SEM [mL × g protein^{-1} × min^{-1}]	Stereoselectivity K_m	Stereoselectivity V_{max}	Stereoselectivity Cl_{int}
OCT3	Metaproterenol-1	245 ± 81	1390 ± 185	5.67 ± 2.63	2.29-fold for 1	2.70-fold for 1**	6.17-fold for 1
	Metaproterenol-2	561 ± 262	516 ± 133	0.92 ± 0.67			
	Milnacipran-1	120 ± 59	355 ± 115	2.96 ± 2.41	2.02-fold for 2	1.12-fold for 2	2.25-fold for 2
	Milnacipran-2	59.4 ± 25.4	396 ± 90	6.67 ± 4.38			
	Oxyphenonium-1	10.4 ± 2.3	225 ± 11	21.8 ± 5.8	1.08-fold for 2	1.63-fold for 1**	1.51-fold for 1
	Oxyphenonium-2	9.6 ± 2.6	138 ± 8	14.4 ± 4.7			
	Pirbuterol-1	241 ± 110	708 ± 145	2.94 ± 1.94	1.00-fold for 1/2	1.13-fold for 2	1.13-fold for 2
	Pirbuterol-2	241 ± 108	803 ± 160	3.33 ± 2.15			
	Sotalol-1	73.9 ± 68.2	61.0 ± 16.1	0.83 ± 0.98	1.14-fold for 1	1.77-fold for 1	2.02-fold for 1
	Sotalol-2	84.2 ± 116	34.4 ± 14.1	0.41 ± 0.73			
	(R)-Tamsulosin	116 ± 101	798 ± 285	6.89 ± 8.50	1.08-fold for *(S)*	1.03-fold for *(R)*	1.05-fold for *(S)*
	(S)-Tamsulosin	107 ± 92	772 ± 263	7.22 ± 8.70			
	Terbutaline-1	233 ± 62	762 ± 89	3.27 ± 1.25	2.04-fold for 2	8.82-fold for 1**	4.31-fold for 1
	Terbutaline-2	114 ± 58	86.3 ± 14.6	0.76 ± 0.51			
	Xamoterol-1	158 ± 50	153 ± 18	0.97 ± 0.42	1.35-fold for 2	1.21-fold for 2	1.64-fold for 2
	Xamoterol-2	117 ± 29	185 ± 15	1.59 ± 0.52			
	(R)-Zolmitriptan	146 ± 101	54.3 ± 16.9	0.37 ± 0.37	11.1-fold for *(S)*	10.2-fold for *(S)****	112-fold for *(S)*
	(S)-Zolmitriptan	13.2 ± 6.0	553 ± 48	41.8 ± 22.5			

SEM, standard error of the mean; asterisks indicate statistical significance of the differences between the two enantiomers (Student's t-test; *$p < 0.05$, **$p < 0.01$, ***$p < 0.001$).

Table S2 HPLC conditions for chiral separation of investigated substances

**Substance**	**HPLC column**	**Mobile phase composition**	**Flow rate** [µL min-1]	**Temperature** [°C]	**Retention time** [min; order of elution]
Amisulpride	Chiralpak CBH	10 mM NH_4Ac, pH 5.8; 10% IPA	300	25	5.35 / 5.74 [S-R]
Carteolol	Chiralpak CBH	10 mM NH_4Ac, pH 5.0; 10% IPA	500	25	3.03 / 5.05 [1-2]
Clidinium	Chiralpak AGP	10 mM NH_4Ac, pH 6.8; 10% IPA	200	25	11.43 / 12.15 [1-2]
Etilefrine	ChirobioticT	20 mM NH_4Ac, pH 4.5; 92% MeOH	400	25	7.49 / 7.87 [1-2]
Formoterol	Chiralpak CBH	10 mM NH_4Ac, pH 5.8; 10% IPA	300	22	6.14 / 6.66 [RR-SS]
Frovatriptan	Chiralpak CBH	10 mM NH_4Ac, pH 5.0; 10% IPA	400	25	5.87 / 7.82 [S-R]
Homatropine	Chiralpak AGP	10 mM NH_4Ac, pH 6.8; 10% IPA	200	25	7.22 / 7.66 [1-2]
Metaproterenol	ChirobioticT	20 mM NH_4Ac, pH 4.5; 92% MeOH	400	25	6.14 / 7.07 [1-2]
Mepenzolate	Chiralpak AGP	10 mM NH_4Ac, pH 6.8; 10% IPA	200	25	10.08 / 10.65 [1-2]
Milnacipran	Chiralpak AGP	10 mM NH_4Ac, pH 6.8; 10% IPA	200	25	8.64 / 8.97 [1-2]
Oxyphenonium	Chiralpak AGP	10 mM NH_4Ac, pH 5.8; 10% IPA	200	25	7.99 / 9.41 [1-2]
Pirbuterol	Chiralpak CBH	10 mM NH_4Ac, pH 5.8; 10% IPA	300	25	3.37 / 3.83 [1-2]
Sotalol	ChirobioticT	20 mM NH_4Ac, pH 4.5; 92% MeOH	400	25	8.90 / 9.48 [1-2]
Tamsulosin	Chiralpak CBH	10 mM NH_4Ac, pH 5.0; 10% IPA	500	25	3.41 / 6.74 [R-S]
Terbutaline	ChirobioticT	20 mM NH_4Ac, pH 4.5; 92% MeOH	400	25	5.89 / 7.13 [1-2]
Xamoterol	Chiralpak CBH	10 mM NH_4Ac, pH 5.8; 10% IPA	300	22	4.72 / 5.84 [1-2]

AGP, α1-glycoprotein; CBH, Cellobiohydrolase; IPA, isopropyl alcohol; MeOH, methanol; NH_4Ac, ammonium acetate;

Table S3 Mobile phase compositions of achiral substance separation

**Substance**	**Flow rate** [µL min-1]	**Retention time** [min]	**Internal standard**
**3% organic additive** (96.9% H_2O, 0.1 % formic acid, 2.6% acetonitrile, 0.4% methanol)			
Choline-d9	300	2.80	-
Ethambutol	300	2.59	Choline-d9
**20% organic additive** (79.9% H_2O, 0.1 % formic acid, 17.2% acetonitrile, 2.8% methanol)			
N-Ethyllidocaine	300	5.29	-
Zolmitriptan	300	3.54	N-Ethyllidocaine
**35% organic additive** (64.9% H_2O, 0.1 % formic acid, 30.0% acetonitrile, 5.0% methanol)			
Aclidinium	400	4.37	Bupivacaine
Bupivacaine	400	2.66	-

Table S4 Mass spectrometry detection parameters

**Substance**	**Mass Q1**[a] [Da]	**Mass Q3**[b] [Da]	**DP**[c] [V]	**CE**[d] [V]	**CXP**[e] [V]
Aclidinium	484.185	262.2 (140.2)	110	45 (72)	18 (10)
Amisulpride	370.2	242.1 (195.8)	90	38 (52)	15 (15)
Bambuterol	368.197	294.2 (72.1)	71	27 (59)	10 (14)
Bupivacaine	289.248	140.1 (84.2)	75	30 (58)	8 (16)
Carteolol	293.219	237.3 (202.1)	63	21 (30)	16 (12)
Choline-d9	113.1	69.1 (66.1)	66	27 (44)	12 (12)
Clidinium	353.179	142 (143.1)	105	43 (45)	8 (10)
Etilefrine	182.124	164 (91)	51	17 (37)	10 (6)
Ethambutol	205.2	116.1 (55.1)	66	21 (45)	6 (10)
Formoterol	345.2	149.1 (121.1)	70	28 (42)	15 (15)
Frovatriptan	244.3	213 (170.1)	56	19 (34)	14 (10)
Homatropine	277.2	142.1 (125.2)	100	40 (33)	12 (12)
Metaproterenol	212.13	152 (107)	56	23 (39)	10 (8)
Mepenzolate	341.2	130 (58)	96	39 (85)	8 (10)
Milnacipran	247.174	230.2 (100.1)	51	17 (27)	14 (8)
N-Ethyllidocaine	264.2	86 (58)	81	36 (64)	16 (11)
Oxyphenonium	348.237	73 (132.2)	96	47 (41)	14 (8)
Pirbuterol	241.3	167.2 (149.1)	65	24 (30)	15 (15)
Proguanil	254.2	170.2 (153.1)	75	24 (40)	10 (10)
Sotalol	273.37	255.1 (133.1)	61	17 (37)	16 (8)
Tamsulosin	409.227	228.1 (271.1)	91	33 (27)	14 (18)
Terbutaline	226.200	152.0 (107)	60	23 (40)	10 (10)
Xamoterol	340.219	253.1 (157)	81	22 (33)	16 (10)
Zolmitriptan	288.000	243 (182)	75	24 (35)	12 (12)

Detection parameters for a second substance-specific mass transition used as qualifier are shown in parentheses. Abbreviations: [a]Q1, first quadrupole; [b]Q3, third quadrupole; [c]DP, declustering potential; [d]CE, collision energy; [e]CXP, collision cell exit potential
